# Supplementary figures and images for: Exploring the Genetic Variability of Gmelina arborea Roxb. in Mexico with Molecular Markers to Establish an Efficient Improvement Program
Source: Plants (Basel). 2025 Jun 19;14(12):1888. doi: 10.3390/plants14121888 (PMC12196935; doi:10.3390/plants14121888)

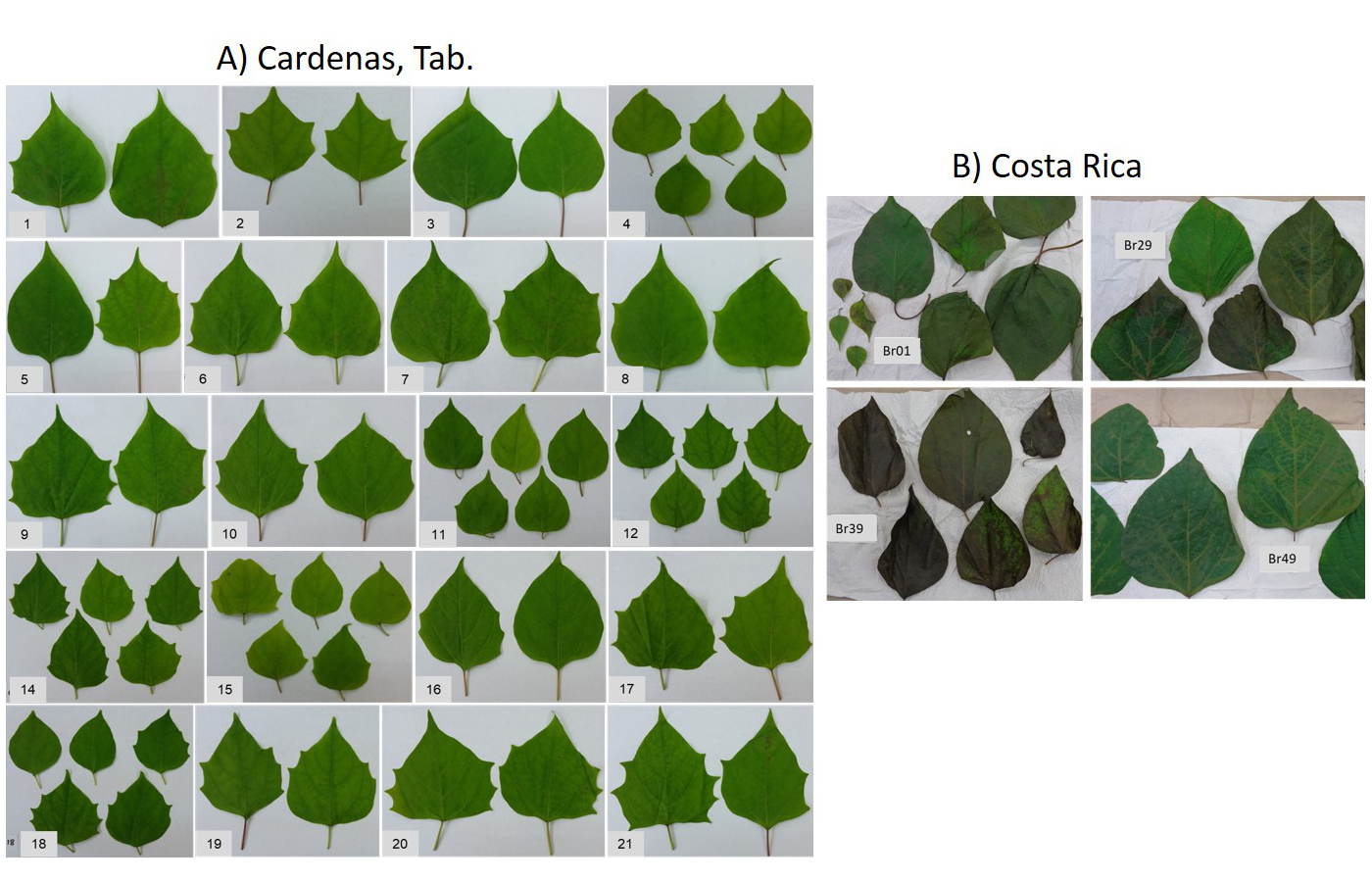

Supplement: Supplementary file 1 [file plants-14-01888-s001.zip › Figure S1.tif]

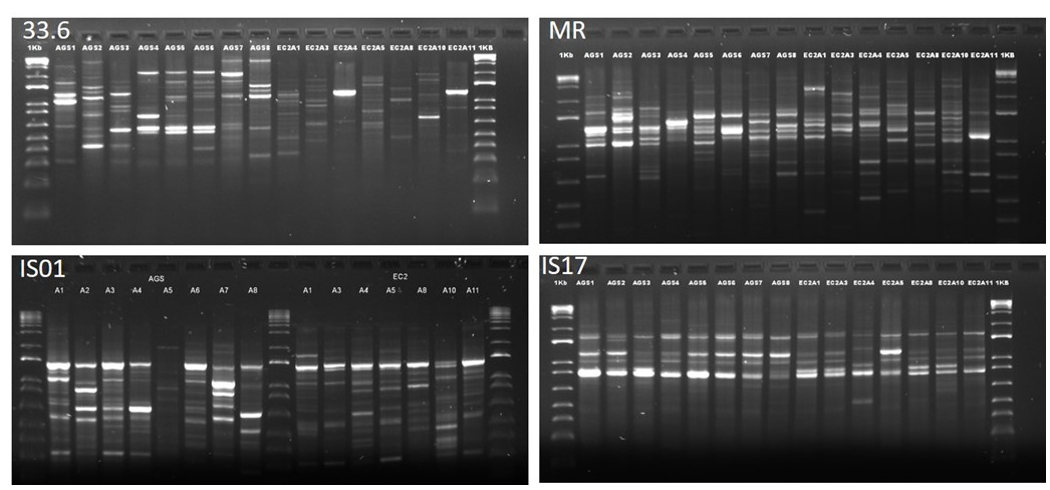

Supplement: Supplementary file 1 [file plants-14-01888-s001.zip › Figure S2.tif]

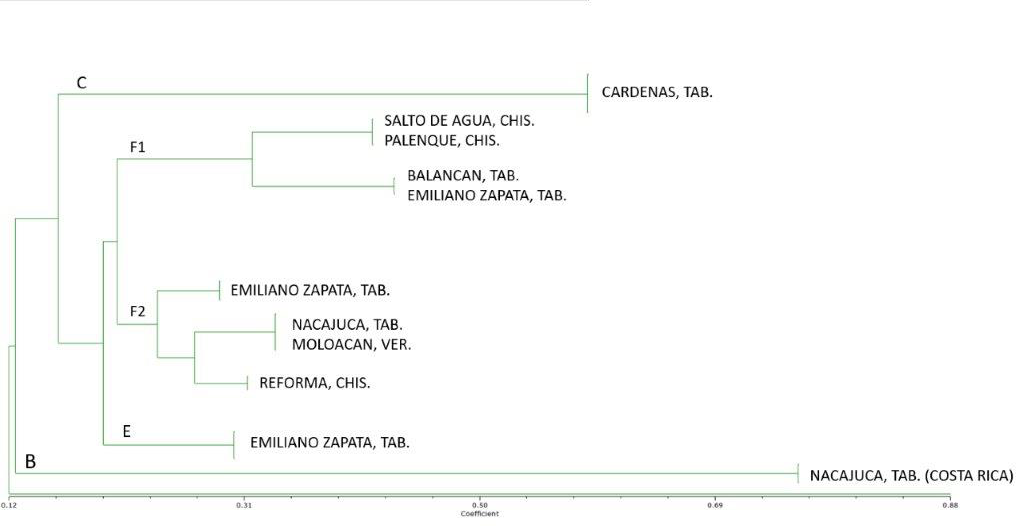

Supplement: Supplementary file 1 [file plants-14-01888-s001.zip › Figure S3.tif]
